# Mechanisms of HIV Transcriptional Regulation and Their Contribution to Latency

**DOI:** 10.1155/2012/614120

**Published:** 2012-06-03

**Authors:** Gillian M. Schiralli Lester, Andrew J. Henderson

**Affiliations:** Section of Infectious Diseases, Department of Medicine, Boston University School of Medicine, Boston, MA 02118, USA

## Abstract

Long-lived latent HIV-infected cells lead to the rebound of virus replication following antiretroviral treatment interruption and present a major barrier to eliminating HIV infection. These latent reservoirs, which include quiescent memory T cells and tissue-resident macrophages, represent a subset of cells with decreased or inactive proviral transcription. HIV proviral transcription is regulated at multiple levels including transcription initiation, polymerase recruitment, transcription elongation, and chromatin organization. How these biochemical processes are coordinated and their potential role in repressing HIV transcription along with establishing and maintaining latency are reviewed.

## 1. Introduction

A critical step in the HIV life cycle is transcriptional regulation of the integrated provirus. Robust transcription assures that sufficient mRNA and genomic RNA are produced for efficient virus assembly and infectivity. Repression of HIV transcription leads to the establishment of HIV latency, which creates repositories for infectious and drug-resistant viruses that reemerge upon treatment failure or interruption [1–4]. The existence of long-lived stable HIV reservoirs was demonstrated by the rebound of virus replication following highly active antiretroviral therapy (HAART) interruption [5–8]. These latent reservoirs, which include quiescent memory T cells, tissue-resident macrophages [9, 10], and potentially hematopoietic stem cells [11], although this is still controversial [12], represent long-lived subsets of cells with decreased or inactive proviral transcription. In general, studies with chronically and acutely infected cells show that mutations in Tat [13, 14], absence of cellular transcription factors [15–18], miRNA machinery [19, 20], and proviral integration into transcriptionally silent sites contribute to postintegration latency [21, 22]. Although there may not be a common mechanism that promotes HIV latency, it is critical to understand the molecular events that establish and maintain latency if strategies to reduce or purge HIV from latent reservoirs are to be devised [9, 23, 24]. HIV transcription is regulated at multiple levels including transcription initiation, polymerase recruitment, transcriptional elongation, and chromatin organization. How these events are coordinated and their role in HIV latency will be reviewed. In particular, mechanisms that contribute to repressing HIV transcription will be highlighted.

## 2. LTR and Transcription Factors

**Figure 1:**
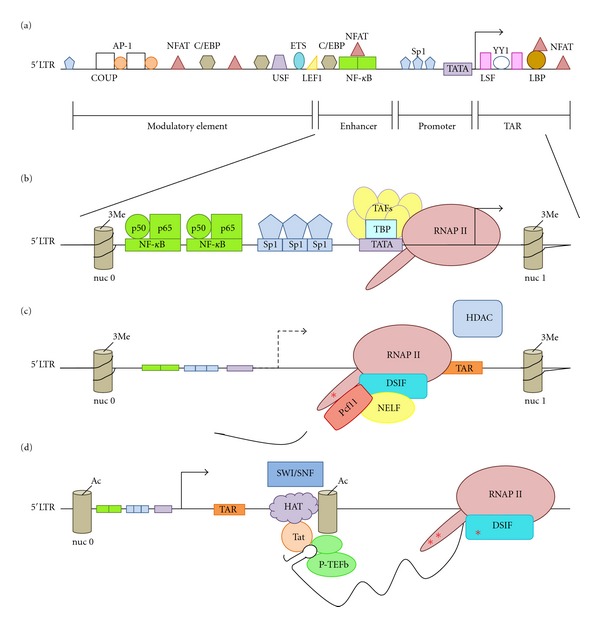
Regulation of HIV transcription initiation and elongation. (a) HIV LTR organization. This only represents a small subset of cis-elements and transcription factors, which bind these sites. (b) Cellular transcription factors are recruited to LTR elements and initiation complex forms at the transcriptional start site. Nucleosomes are posttranslationally modified favoring a condensed chromatin structure that impedes RNAP II transcriptional elongation. (c) RNAP II processes a short distance downstream from the transcriptional start site when DSIF and NELF induce a pause in transcription. Pcf11 reinforces this block in elongation by prematurely terminating the transcription of the short nascent RNA product. HDAC recruitment to the paused complex reinforces a transcriptionally repressed chromatin state. The red asterisk depicts phosphorylation of RNAP II CTD at serine 5 position. (d) RNAP II elongation complex is released from the transcriptional pause by the recruitment of P-TEFb, which mediates hyperphosphorylation of the CTD at serine 2 position and phosphorylation of DSIF, which induces NELF disassociation from the complex (red asterisks indicate key phosphorylation events). The recruitment of chromatin remodeling machinery such as HATs and PBAF SWI/SNF facilitates acetylation of nucleosomes, which displaces the blocking nucleosome and supports transcription elongation.

Although viral accessory proteins, such as Vpr, and putative elements within the HIV provirus genome may influence HIV transcription [25, 26], the dominant HIV transcriptional regulatory element is the 5′ long terminal repeat (LTR). The HIV LTR is often divided into four functional elements: the Tat activating region (TAR), which in the context of the nascent RNA forms an RNA stem loop structure that binds the virus-encoded transactivator Tat; the promoter, the enhancer, and the negative/modulatory regulatory element (Figure 1(a)). The promoter, enhancer and modulatory elements recruit a plethora of tissue specific and ubiquitously expressed host-transcription factors that function as activators, repressors, or adapter proteins (see references for detailed reviews [27–29]). AP-1, Sp1, and NF-*κ*B are required for efficient basal and induced HIV transcription and replication [27–32]. One major check-point in the control of HIV transcription is the availability of critical transcription factors. Several inducible transcription factors have been identified in T cell and monocytic cell lines that transactivate the HIV LTR, including AP-1 [30, 33, 34], C/EBPb [35, 36], NFAT [37–39], Ets/PU.1 [40, 41], and TCF/LEF-1 [42, 43] to cite a select few. A classic example of transcription factor availability regulating function is the binding of NF-*κ*B to sites within the HIV LTR [44]. Upon cell activation, the p65 subunit is released from the I*κ*B inhibitory complex, dimerizes with the p50 NF-*κ*B subunit, and translocates from the cytoplasm to the nucleus, where it binds the NF-*κ*B sites in HIV LTR to mediate efficient transcription [17]. However, sequestering p65 in the cytoplasm through its interaction with I*κ*B limits the availability of active NF-*κ*B in the nucleus and HIV provirus transcription. Furthermore, this transcriptional repression is reinforced by p50-p50 homodimers binding the NF-*κ*B sites and recruiting histone deacetylase complexes (HDACs), which promote a repressive chromatin state [18]. In addition, there have been reports of several cellular transcription factors that repress transcription in the context of HIV latency. They include but are not limited to, the ubiquitous factors LSF-1, YY-1 [45, 46]; Sp1 and the bHLH-zipper proto-oncogene c-Myc [47]; CTIP-2/Bcll11b, a COUP-TF interacting protein expressed in the central nervous system that interacts with Sp1 [48, 49]; CBF-1, an effector of Notch signaling that is regulated during T cell activation [50]; ligand-activated nuclear receptors [51]; FBI-1, a POZ domain, Kruppel-type zinc finger [52]. Which combination of these factors potentially establishes latency in specific cellular subsets is a critical question that needs to be addressed.

## 3. Chromatin and HIV Transcription

One function of transcription factors is to recruit complexes that influence chromatin organization. For example, transcriptional activators such as NF-*κ*B, NFAT, and C/EBP*β* recruit histone acetyltransferases (HATs) that modify key lysines on histone 3 and histone 4 [10, 24, 44, 53–56]. Histone acetylation, which is associated with active transcription, results in an open or accessible DNA conformation that is more amenable to the binding of additional transcriptional activators, initiation factors, and RNA polymerase II (RNAP II). SWI/SNF complexes and demethylases are recruited to promoters and enhancers by transcription factors and coactivators to remodel nucleosomes, especially around the promoter and transcriptional start sites of genes, resulting in the induction of transcription. The chromatin organization of the HIV LTR has been studied in detail (reviewed in [55–57]). The HIV LTR is flanked by two positioned nucleosomes, nuc-0 at the 5′ end of the LTR and nuc-1 that is juxtaposed to the transcriptional start site (Figure 1(b)). Induction of HIV transcription correlates with histone acetylation, recruitment of HATs [53, 58–60], PBAF containing SWI/SNF complexes [61–64], and displacement of nuc-1 [57, 61, 63–67]. These posttranscriptional modifications to the chromatin state are associated with HIV transcription.

Reversing the posttranslational modifications associated with transcriptional activation is accomplished by recruiting SWI/SNF complexes, HDACs, and/or methyltransferases, which catalyze histone trimethylation. These inhibitory modifications are proposed to contribute to a more condensed chromatin structure which impedes RNAP II processivity and transcription elongation [68, 69]. For SWI/SNF there are at least two distinctive complexes that have been described, PBAF which has been associated with transcriptional activation and BAF which has been implicated in the establishment and maintenance of HIV latency [62, 64]. Class I and II HDACs [54, 70], the methyltransferases Suv39H1, Zeste 2, and heterochromatin protein 1 (hp-1) [71, 72] have been implicated in mediating the deacetylation and trimethylation of nuc-1 and the repression of HIV transcriptional elongation. Long term repression of transcription can be reinforced by additional epigenetic changes including DNA methylation [55, 73]. In summary, posttranslational modifications of chromatin have been linked to the maintenance of latent viral reservoirs.

## 4. Transcriptional Interference

Although epigenetic events, such as restrictive positioned nucleosomes or DNA methylation, limit HIV transcription recent studies examining proviral integration sites have highlighted the need to consider additional models to explain repression of HIV transcription. Initial experiments by the Bushman laboratory [74–77] in which proviral integration sites in cells that were latently infected with HIV were sequenced indicated that silenced HIV preferentially integrated into transcriptionally active host genes. Similar findings were obtained in infection models with cell lines [77–80] and primary cells, as well as resting CD4 cells from patients either untreated or undergoing HAART [79, 81]. These findings indicate that active neighboring promoters are directly repressing or transcriptionally interfering with the HIV LTR [78, 80, 82]. Transcriptional interference is defined as the suppression of one transcription unit by another neighboring cis-element [83]. Suggested mechanisms that lead to interference of the HIV LTR include the adjacent promoters competing for or displacing the components of transcription initiation complexes, or collisions between transcription elongation complexes moving in opposite directions [83–88]. Although there may be a potential role for chromatin-associated factors in maintaining transcriptional interference [89], other reports from the literature would predict that there are additional critical repressive checkpoints that contribute to HIV latency [78, 82].

## 5. Transcriptional Elongation

Transcription factors assist with the recruitment of the general basal factors, which include the RNAP II itself, TFIID (TATA binding protein or TBP), and the TBP-associated factors (TAFs), TFIIA, TFIIB, TFIIE, TFIIF, and TFIIH, to assemble the core promoter complex and assure proper positioning of the RNAP II at the transcriptional start site (Figure 1(b)). General transcription factors, such as TFIIH, have been implicated as playing a critical role in HIV transcription at times of low Tat expression [90]. However, recently, the concept of a “core” promoter has been challenged by the discovery of tissue-specific TAFs and unique preinitiation complexes [91] favoring models in which the factors found at core promoters and the RNAP II are diverse and dynamic. For example, RNAP II associated protein, Gdown1, competes with TFIIF for RNAP II, therefore inhibiting transcription and promoting the assembly of a paused RNAP II complex [92, 93]. Whether the complexity associated with RNAP II recruitment and assembly reflects cell type and cell-cycle-specific requirements for HIV transcription is just starting to be investigated. However, it has been shown that Tat can influence the recruitment of TBP and associated TAFs [94] suggesting that these early transcriptional complexes are regulated by HIV infection.

Control of transcription elongation is a critical checkpoint in the regulation of a number of genes including c-myc, c-fms, hsp-70, Jun B, and HIV [95–99] and is dependent on the coordination of RNAP II activity, premature transcription termination, and chromatin structure [100]. Furthermore, several genome-wide studies with multiple organisms mapping RNAP II location have shown that 20–30% of genes have enriched RNAP II density at the 5′ end of the gene relative to the body of the gene. This was discovered for genes with both detectable or undetectable transcription [101–104] suggesting that post-RNAP II recruitment and transcriptional elongation represents a key rate-limiting transcriptional checkpoint for gene expression [105]. The interplay between the negative elongation factors, negative elongation factor (NELF) and DRB sensitivity-inducing factor (DSIF), and positive elongation factors, such as P-TEFb [106], sets this checkpoint. NELF and DSIF associate with the early elongation complex and inhibit RNAP II processivity, possibly by interfering with the extrusion of the nascent transcript from the elongation complex [107]. P-TEFb, which is composed of a regulatory Cyclin T1 (CycT1) subunit and an enzymatic Cyclin-dependent kinase 9 (Cdk9) subunit, alleviates transcriptional repression by phosphorylating one or more of the components in this complex as well as the carboxy terminal domain (CTD) of RNAP II at serine 2 leading to the active engagement of RNAP II in transcription elongation [108–112]. Phosphorylation of DSIF converts DSIF from a negative to a positive elongation factor [106], whereas phosphorylation of NELF by P-TEFb reduces the ability of NELF to associate with RNA [113]. Notably, NELF dissociates from the elongation complex when the complex is transcribing the DNA *in vivo* suggesting that NELF primarily functions as an inhibitor of elongation [114] (Figure 1).

P-TEFb is a general transcription factor, which is required for efficient expression of the majority of cellular genes, and its availability and activity is carefully regulated to allow for changes in global transcriptional demand [115–117]. The regulation of P-TEFb is complex and employs multiple transcriptional and posttranslational strategies that may impact HIV transcription as well as overall cellular gene expression. One mechanism that limits P-TEFb is its association with the 7SK complex, which includes 7SK RNA, HEXIM1, HEXIM2, MePCE, and LARP7 [55, 115–117]. Release of P-TEFb from this complex during T cell activation favors enhanced HIV transcription. Furthermore, recent biochemical profiling has indicated that there are multiple P-TEFb complexes that include association with other coactivators including Brd4 [118–120], SKIP [121, 122], and components of the super elongation complex [116, 123, 124]. Although the significance of these different complexes with regard to HIV latency is still being explored, it is tempting to speculate that these additional cofactors could couple transcription elongation with other processes that influence gene expression including chromatin organization and splicing. P-TEFb activity is also regulated by phosphorylation and dephosyphorylation in the T-loop domain of Cdk9. Although the kinase responsible for Cdk9 posttranslational modification has not been reported, several phosphatases, PPM1, PP1, PP2A, PP2B have been implicated in regulating P-TEFb and HIV transcription [125–129]. Finally, P-TEFb activity is in part regulated by expression of CycT1, which is regulated at a transcriptional level in macrophages and CD4+ T cells [130].

Recruitment of P-TEFb to the HIV LTR is a critical step for transcriptional activation and this is the primary function of the viral transcriptional activator Tat. Furthermore, NELF and DSIF, which are necessary for pausing RNAP II, are both bound to the HIV LTR after initiation of viral transcription [110, 113, 131]. The NELF E subunit, which has an RNA binding domain, has been shown to bind the HIV-TAR element and inhibit Tat transactivation [113, 132]. Diminishing the Spt5 subunit of DSIF decreases HIV replication [110], whereas decreasing NELF expression releases paused polymerases on the HIV LTR and induces HIV transcription elongation in cell line models for transcriptional latency. In addition, depleting NELF induced histone acetylation and displacement of the positioned nucleosome, hinting that transcription elongation and chromatin remodeling maybe coupled processes [131].

In the context of HIV, RNAP II processivity and transcriptional elongation are highly regulated events as suggested by the accumulation of short transcripts in the cytoplasm in HIV-infected cells [96–98, 133]. Under conditions that inhibit transcription elongation, RNAP II is prone to premature termination which reenforces the block in RNAP II processivity and the accumulation of short transcripts observed in cells that have repressed HIV provirus. One possibility for this is that a termination complex is recruited to RNAP II, which destabilizes the nonprocessive RNAP II complex similar to 3′ end processing of mRNA and transcription termination. Only two proteins are known that have the capacity to dissociate RNAP II from the DNA template: TTF2, which dissociates the elongation complex in an ATP-dependent manner during chromosome condensation of the M-phase of the cell cycle [134] and Pcf11, which is involved in 3′ end processing of mRNA and transcription termination of protein-encoding genes [135, 136]. Pcf11 has been demonstrated to dissociate transcriptionally engaged RNAP II from DNA, indicating a pivotal role in termination [137–139]. Recent reports show that Pcf11 binds to the HIV LTR and represses HIV transcription in cell line models for HIV latency [140]. Pcf11 may be recruited to the LTR by the paused RNAP II complex. In summary, HIV transcriptional elongation is limited by multiple mechanisms that include the availability of P-TEFb, processiveness of the RNAP II complex, and premature termination (Figure 1(c)).

## 6. Tat

The presence of a blocking nucleosome and the role of pausing and premature termination would indicate that transcriptional elongation presents a major checkpoint to HIV transcription. HIV overcomes this limitation through the function of the virally encoded transcriptional activator Tat. Tat potently activates HIV gene expression by facilitating the recruitment of P-TEFb to the HIV LTR. Tat binds the RNA stem loop structure formed by the TAR element and recruits P-TEFb through its interaction with the CycT1 subunit [141]. The Tat-P-TEFb interaction brings active Cdk9 into the proximity of the paused RNAP II complex. P-TEFb phosphorylates the CTD domain of RNAP II as well as NELF and DSIF, inducing RNAP II processivity and transcriptional elongation. In addition to directly targeting the paused RNAP II complex Tat recruits chromatin remodeling factors such as SWI/SNF complexes Brm and/or Brg-1 [63, 64, 142] as well as HATs, p300/CBP, P/CAF and GCN5 that can promote transcriptional activation through post-translational modification of histones and the remodeling of the positioned nuc-1 [59, 63]. Thus, Tat is positioned to play a critical role in coordinating transcriptional elongation and chromatin remodeling to assure efficient HIV transcription. The transactivation of Tat couples HIV transcriptional elongation along with chromatin remodeling [21, 67] (Figure 1(d)).

Tat activity is regulated at multiple levels including transcription and posttranslational modification [143]. Tat transcription is regulated by the HIV LTR and if repressed, limited Tat will be expressed. Minimal Tat function, either due to lack of cellular factors or mutation to the Tat-TAR axis, favors repression of HIV transcription and latency [55, 143]. In addition, stochastic fluctuations in Tat transcription have been shown to overcome initial repression and induce efficient transcription elongation [144]. Post-translational modifications of Tat have been demonstrated to modulate its interactions with TAR, P-TEFb, and chromatin-remodeling complexes to assure the transactivation of Tat even under limiting conditions [145]. In particular, Tat is subject to a dynamic sequential methylation/demethylation and acetylation/deacetylation cycles. Monomethylation of lysine 51 (K51) by Set7/9/KMT7 enhances Tat binding to the TAR, whereas demethylation by LSD1/KDM1/CoREST and acetylation of neighboring lysine 50 (K50) mediated by p300/KAT3B favor the dissociation of Tat from TAR and P-TEFb [146–150]. SIRT1, the class III nicotinamide adenine dinucleotide-dependent class III protein, deacetylates Tat and represses its activity [149]. The methyltransferase, demethylase HDACs and HATS that control HIV Tat function are attractive therapeutic targets [150].

## 7. Conclusion and Implications

Studies using a variety of cell lines [16, 22, 151] and primary cell systems [37, 152, 153] have provided insights into the complexity of HIV transcription and the appreciation that multiple mechanisms contribute to latency [154]. Furthermore, these studies have suggested that therapeutic strategies targeting transcription may be used to purge HIV from different cellular reservoirs. Attempts to activate repressed proviral transcription present several unique challenges including the lack of a single or common event in establishment and maintenance of latency, and most factors that limit HIV transcription are general transcriptional regulators and cofactors, which are necessary for normal gene expression. Compounds that target RNAP II, P-TEFb, and chromatin remodeling factors will likely be toxic, lack specificity, and have a global impact on gene expression. The challenges that exist in translating our general understanding of HIV transcription into a viable therapeutic approach are highlighted by the recent clinical trials with HDAC inhibitors. Based on the strong evidence from cell line models of HIV latency, which showed that overcoming the repressive effects of chromatin induces HIV transcription, it was hypothesized that HDAC inhibitors could be a useful tool in purging HIV from latently infected cells [155]. Initial experiments using the HDAC inhibitor valproic acid with primary cells from HIV-positive patients were encouraging [156–158]; however, follow-up studies and a recent clinical trial have shown that valproic acid had a minimal impact on the low level of virema in the peripheral blood of ART patients [159–163]. Although these results might be viewed as discouraging, next-generation HDAC inhibitors [164, 165] in combination with other potential treatments such as methyltransferase inhibitors [166] as well as newly identified compounds discovered in recent screens [89, 153], which target HIV transcription but only partially activate T cells, may be efficacious. As we screen and develop new compounds, it will be critical to assure that they are active in multiple *in vitro* and *in vivo *models of latency to assure that the broad range of potential mechanisms that influence HIV transcription and latency are targeted [154].
